# Supplementary material for: Selective Reflection of Light in Glassforming Ternary Liquid Crystalline Mixtures
Source: J Phys Chem B. 2026 Apr 27;130(18):4951–9. doi: 10.1021/acs.jpcb.6c01650 (PMC13308994; doi:10.1021/acs.jpcb.6c01650)
Supplement: Supplementary file 1 [file jp6c01650_si_001.pdf]

Selective Reflection of Light in Glassforming Ternary Liquid Crystalline Mixtures

Aleksandra Deptuch [a,*], Zuzanna Zając [b], Marcin Piwowarczyk [a], Anna Drzewicz [a], Marcin Kozieł [c], Magdalena Urbańska [d], Ewa Juszyńska-Gałązka [a,e]

[a] Institute of Nuclear Physics, Polish Academy of Sciences, Radzikowskiego 152, PL-31342 Kraków, Poland

[b] Faculty of Materials Science and Ceramics, AGH University of Cracow, PL-30059 Kraków, Mickiewicza 30, Poland

[c] Faculty of Chemistry, Jagiellonian University, Gronostajowa 2, PL-30387, Kraków, Poland

[d] Institute of Chemistry, Military University of Technology, Kaliskiego 2, PL-00908 Warsaw, Poland

[e] Research Center for Thermal and Entropic Science, Graduate School of Science, Osaka University, 560-0043 Osaka, Japan

*corresponding author, aleksandra.deptuch@ifj.edu.pl

Supporting Information

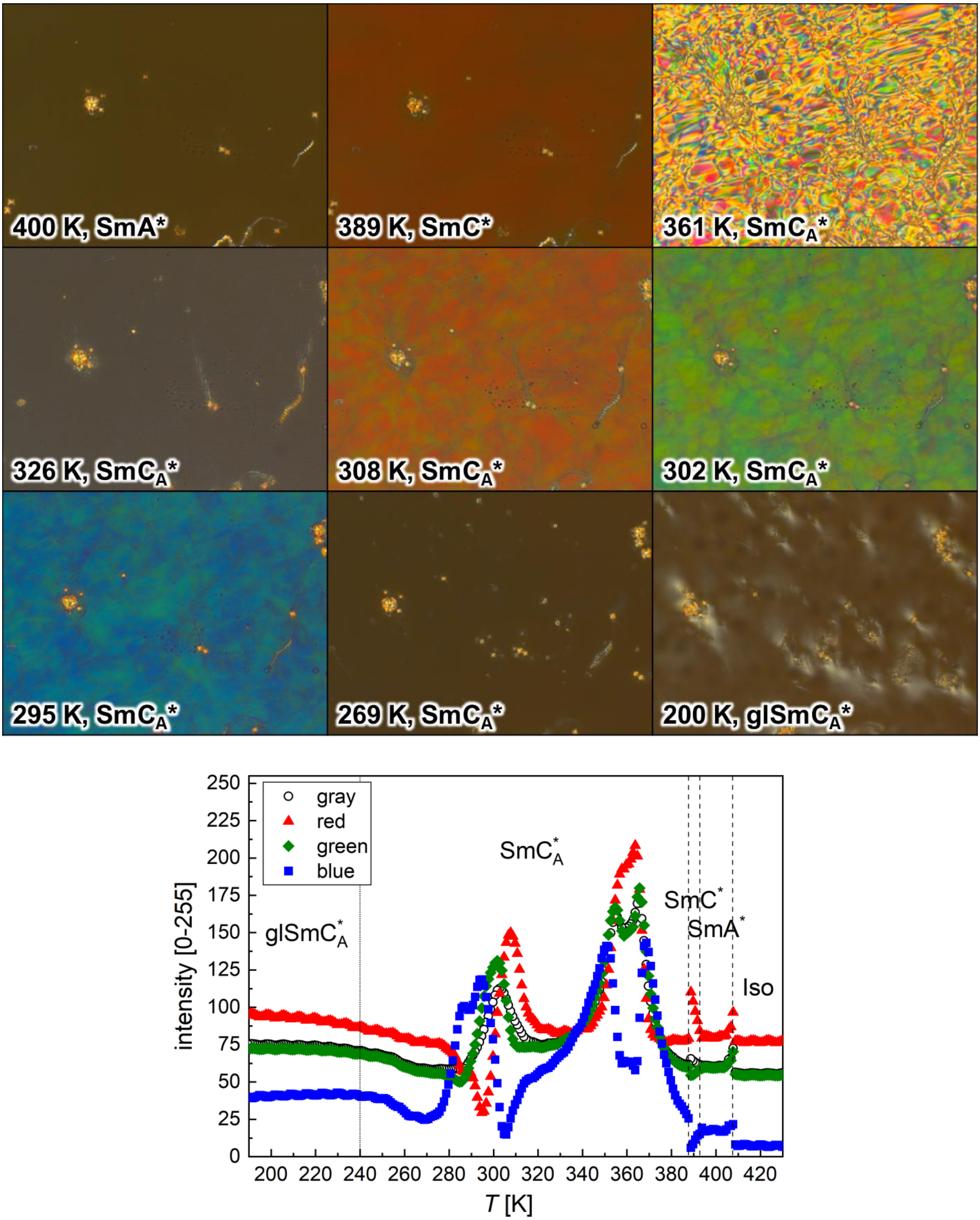

Figure S1. Representative POM textures (622 × 466 μm^2) of MIX5HFHH6 collected at the 10 K/min cooling rate in the transmission mode as well as the red, green, blue components and weighted total intensity of each texture. The glass transition temperature is based on DSC results.

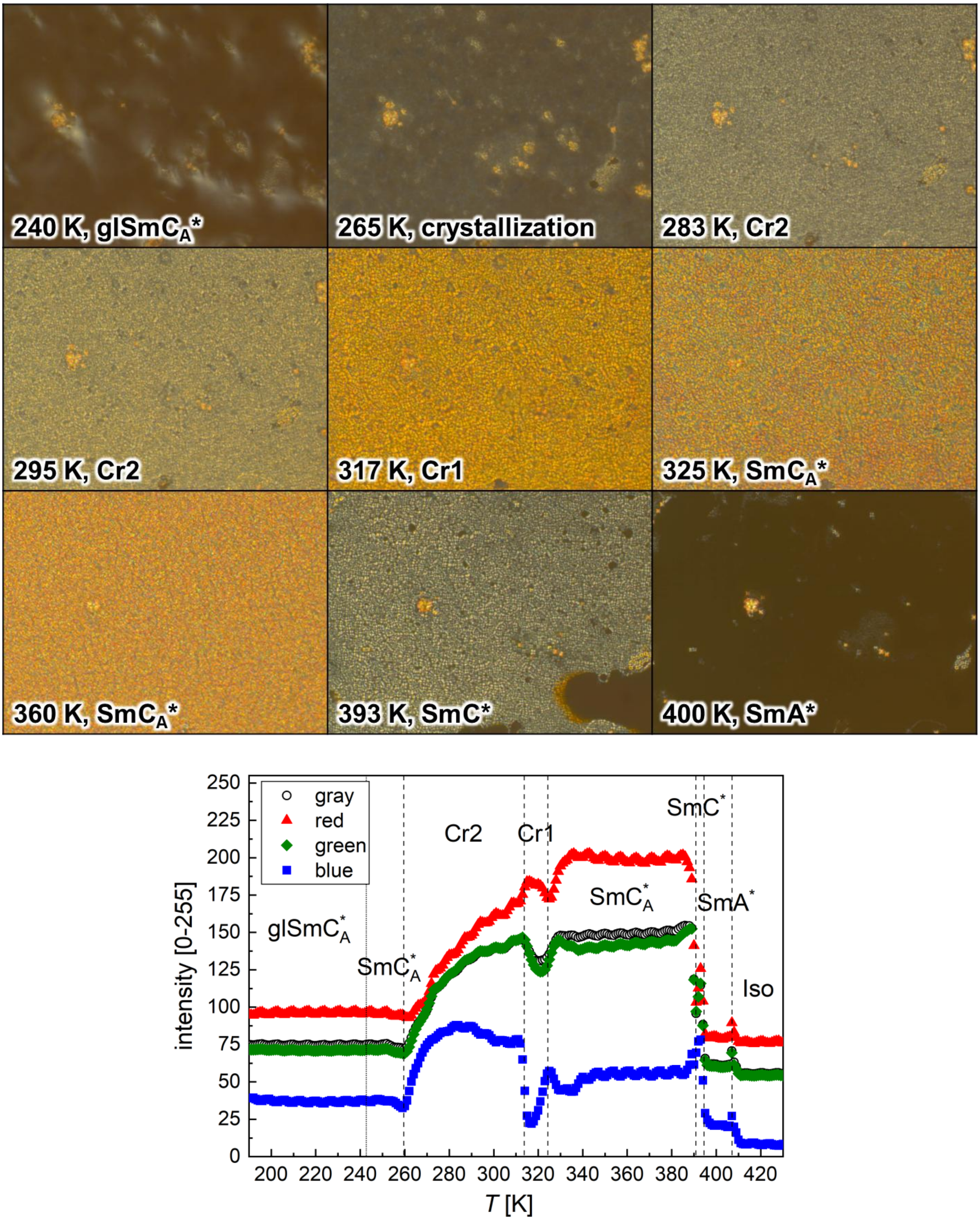

Figure S2. Representative POM textures (622 × 466 μm^2) of MIX5HFHH6 collected at the 10 K/min heating rate in the transmission mode as well as the red, green, blue components and weighted total intensity of each texture. The glass transition temperature is based on DSC results.

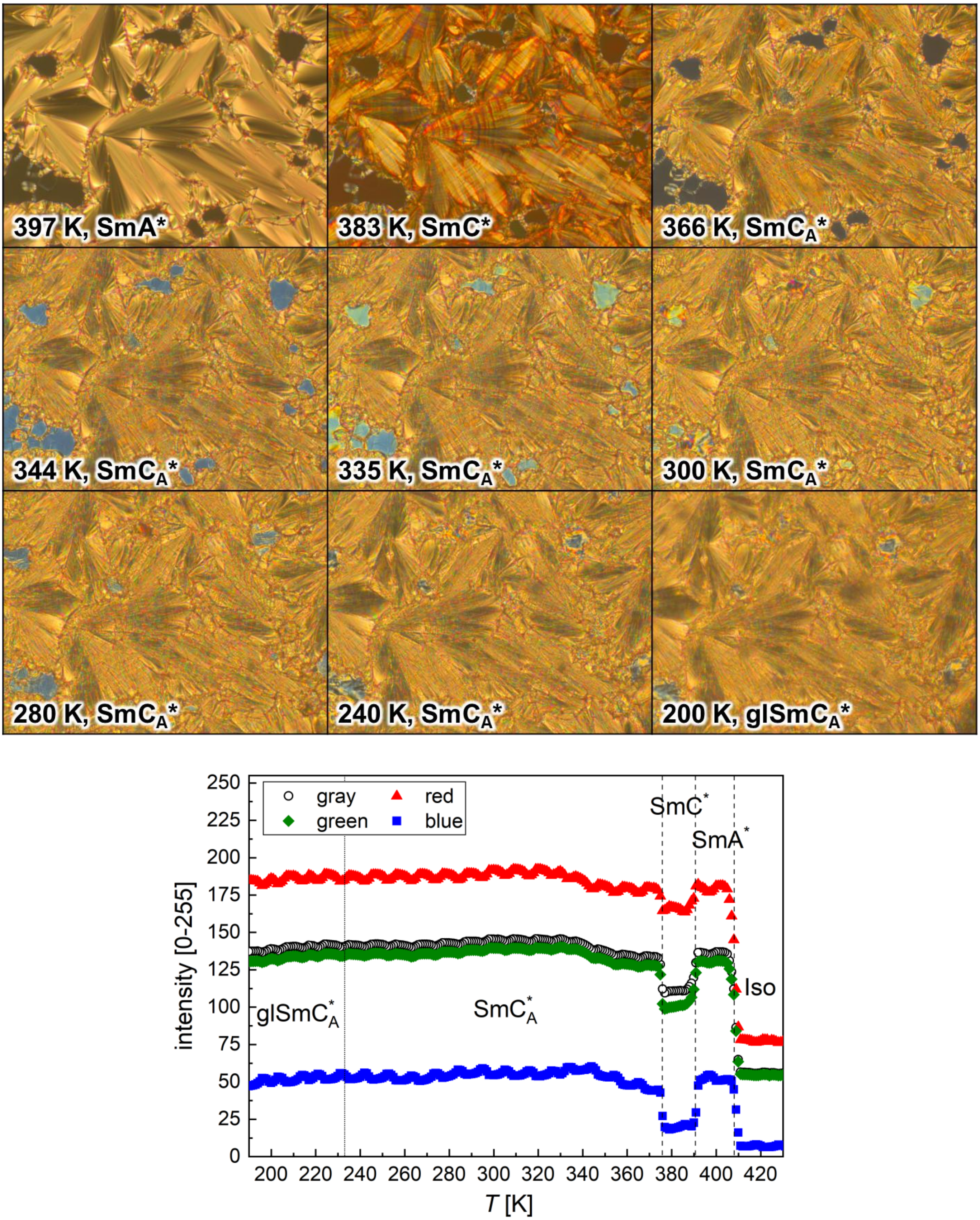

Figure S3. Representative POM textures (622 × 466 μm^2) of MIX6HFHH6 collected at the 10 K/min cooling rate in the transmission mode as well as the red, green, blue components and weighted total intensity of each texture. The glass transition temperature is based on DSC results.

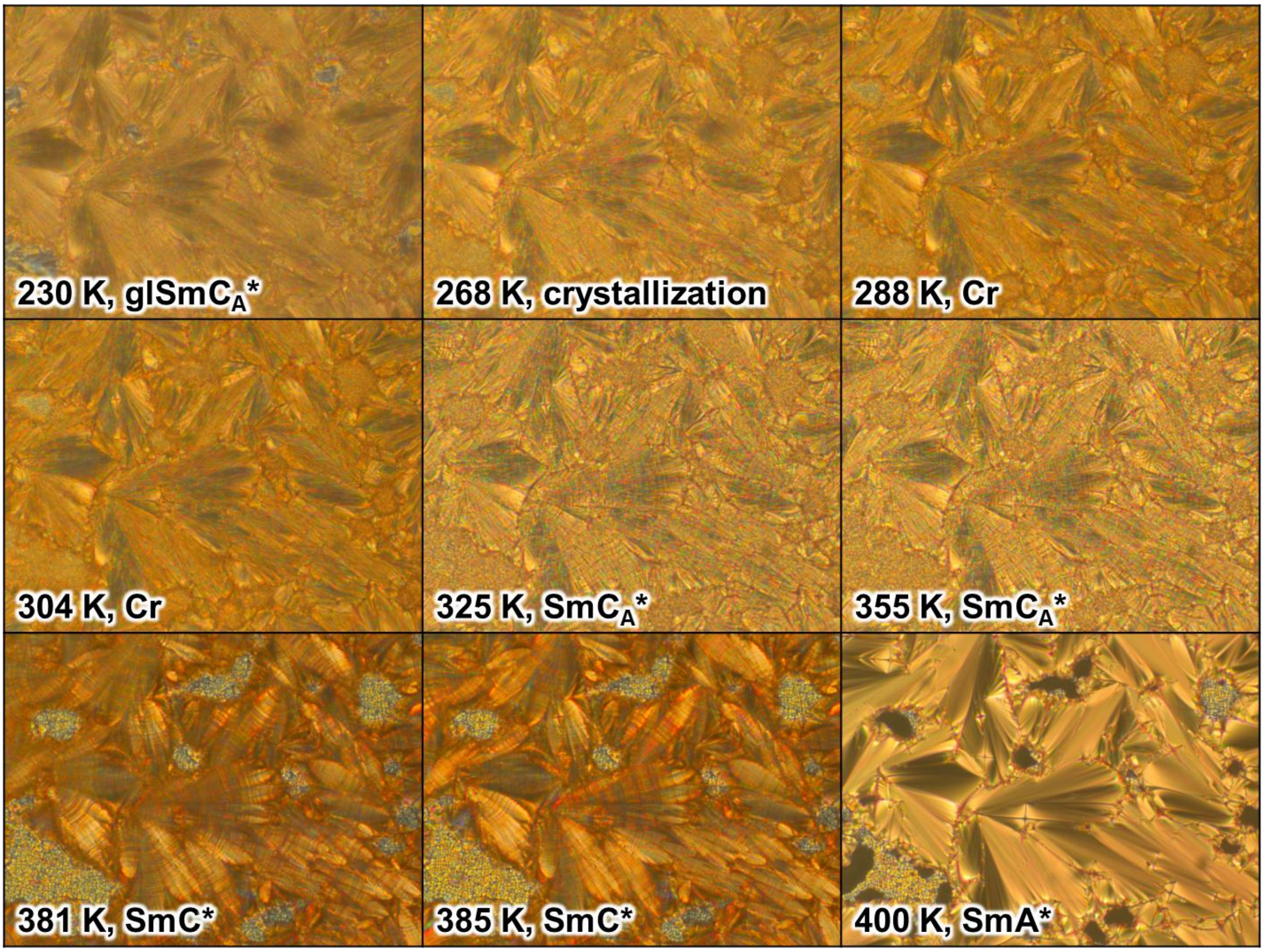

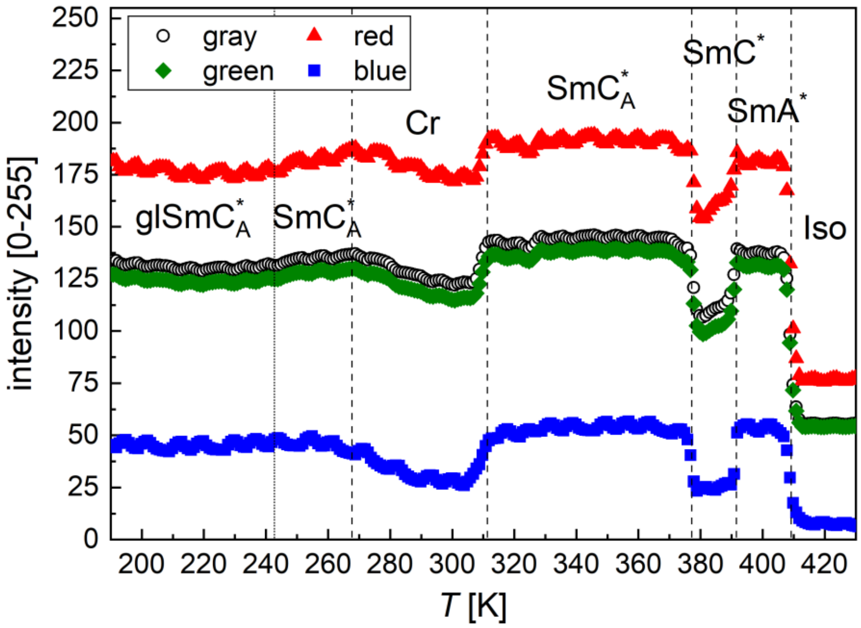

Figure S4. Representative POM textures (622 × 466 μm^2) of MIX6HFHH6 collected at the 10 K/min heating rate in the transmission mode as well as the red, green, blue components and weighted total intensity of each texture. The glass transition temperature is based on DSC results.

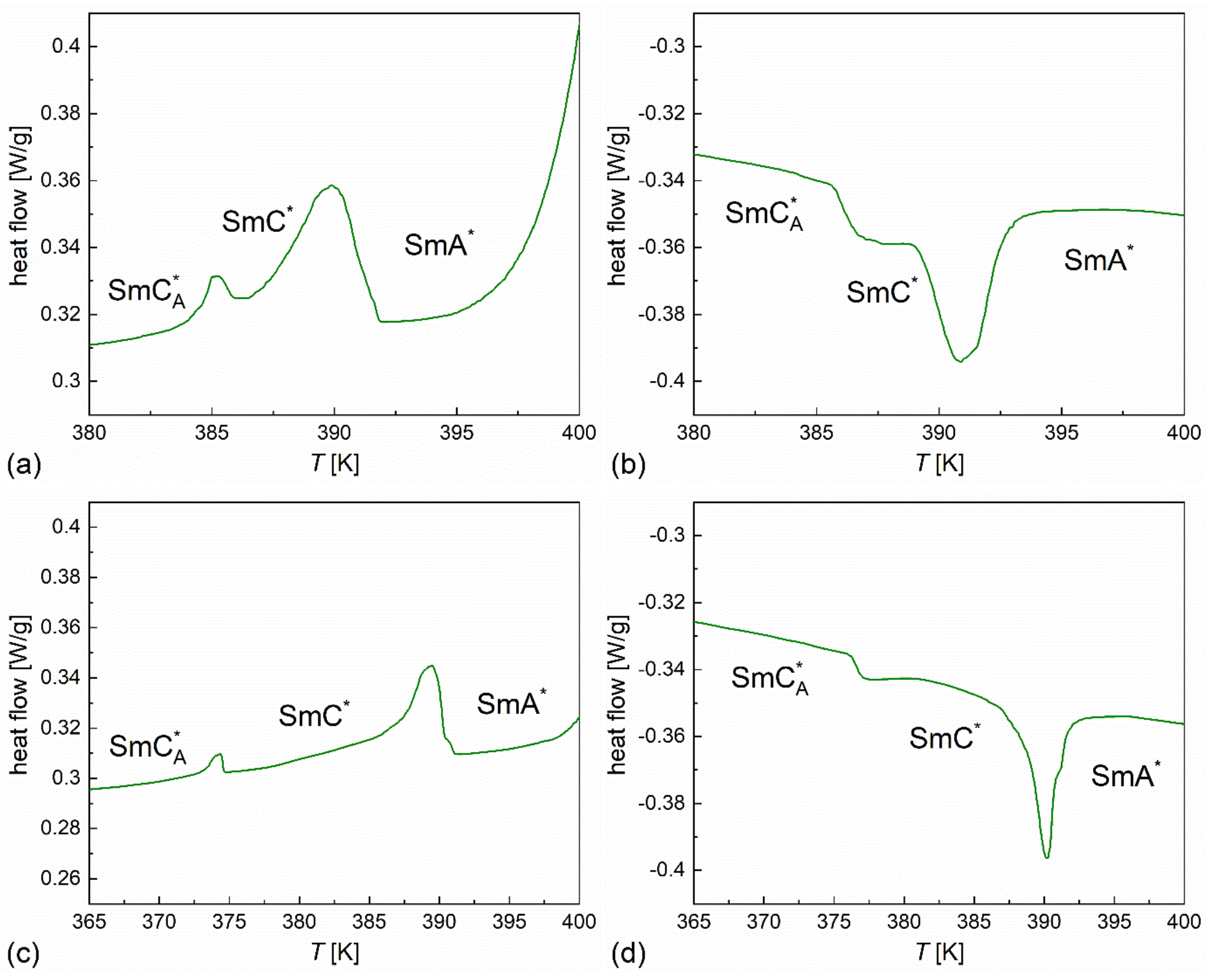

Figure S5. Close-up to transitions between smectic phases in DSC thermograms of MIX5HFHH6 at cooling (a) and heating (b), and of MIX6HFHH6 at cooling (c) and heating (d) at 10 K/min.

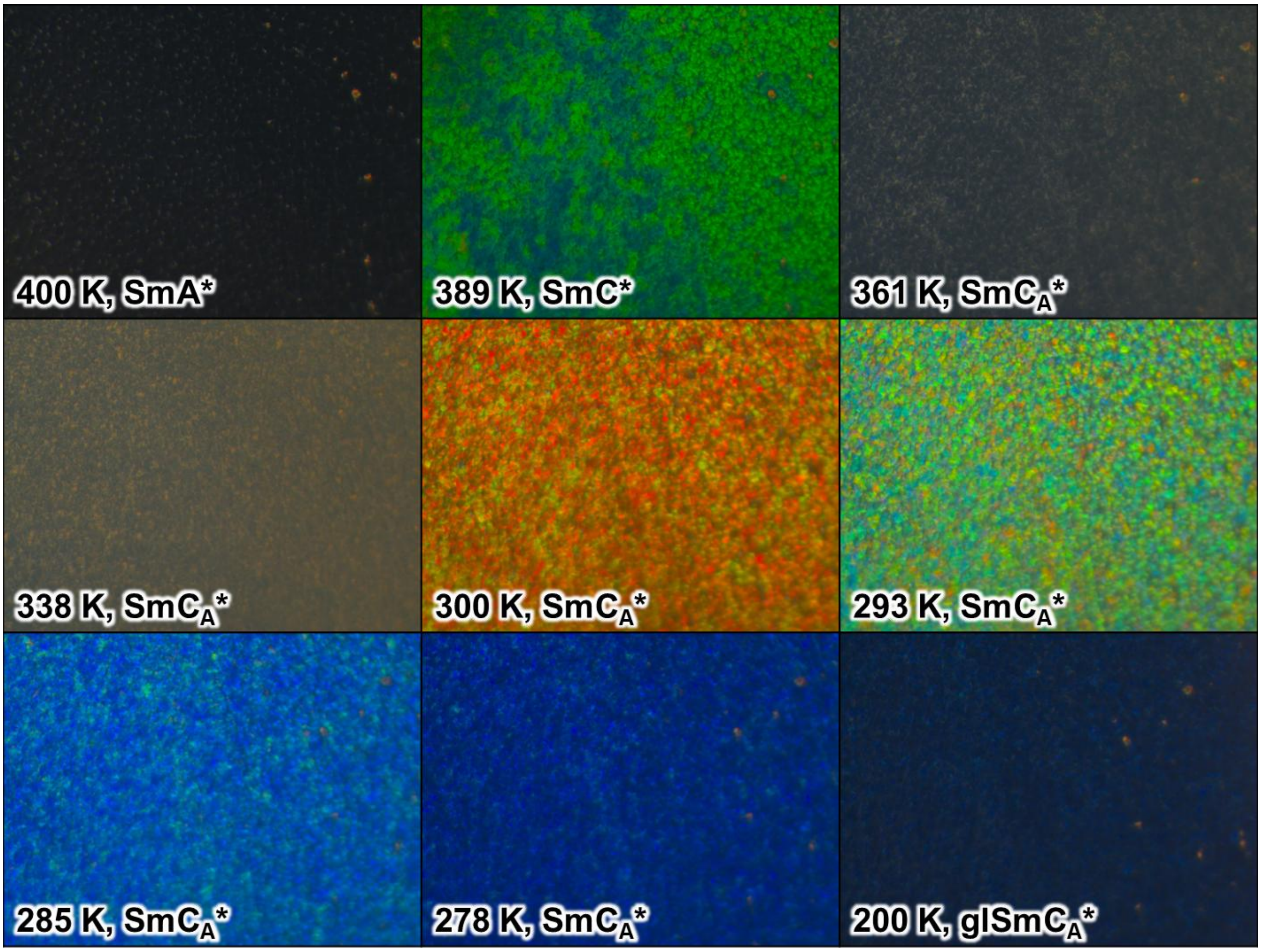

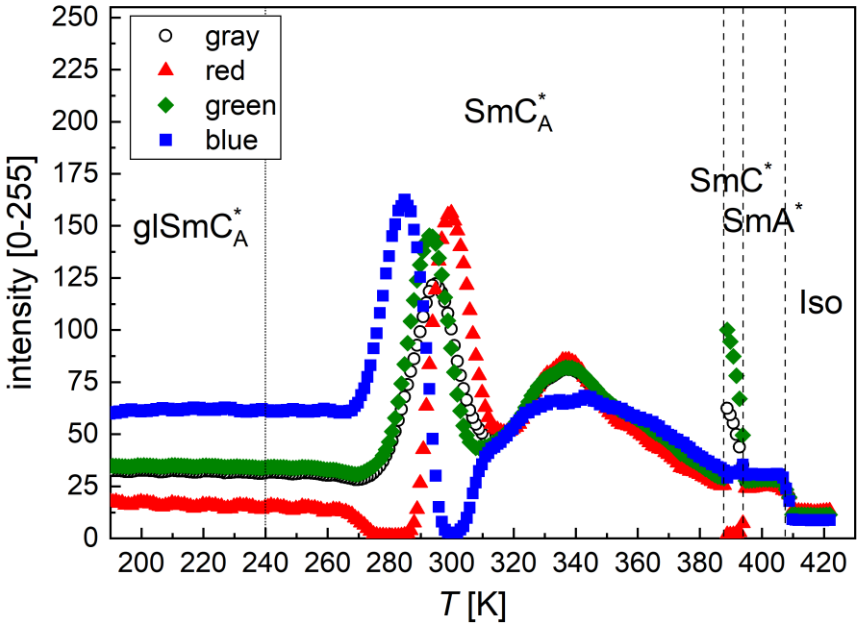

Figure S6. Representative POM textures (622 × 466 μm^2) of MIX5HFHH6 collected at the 10 K/min cooling rate in the reflection mode as well as the red, green, blue components and weighted total intensity of each texture. The glass transition temperature is based on DSC results.

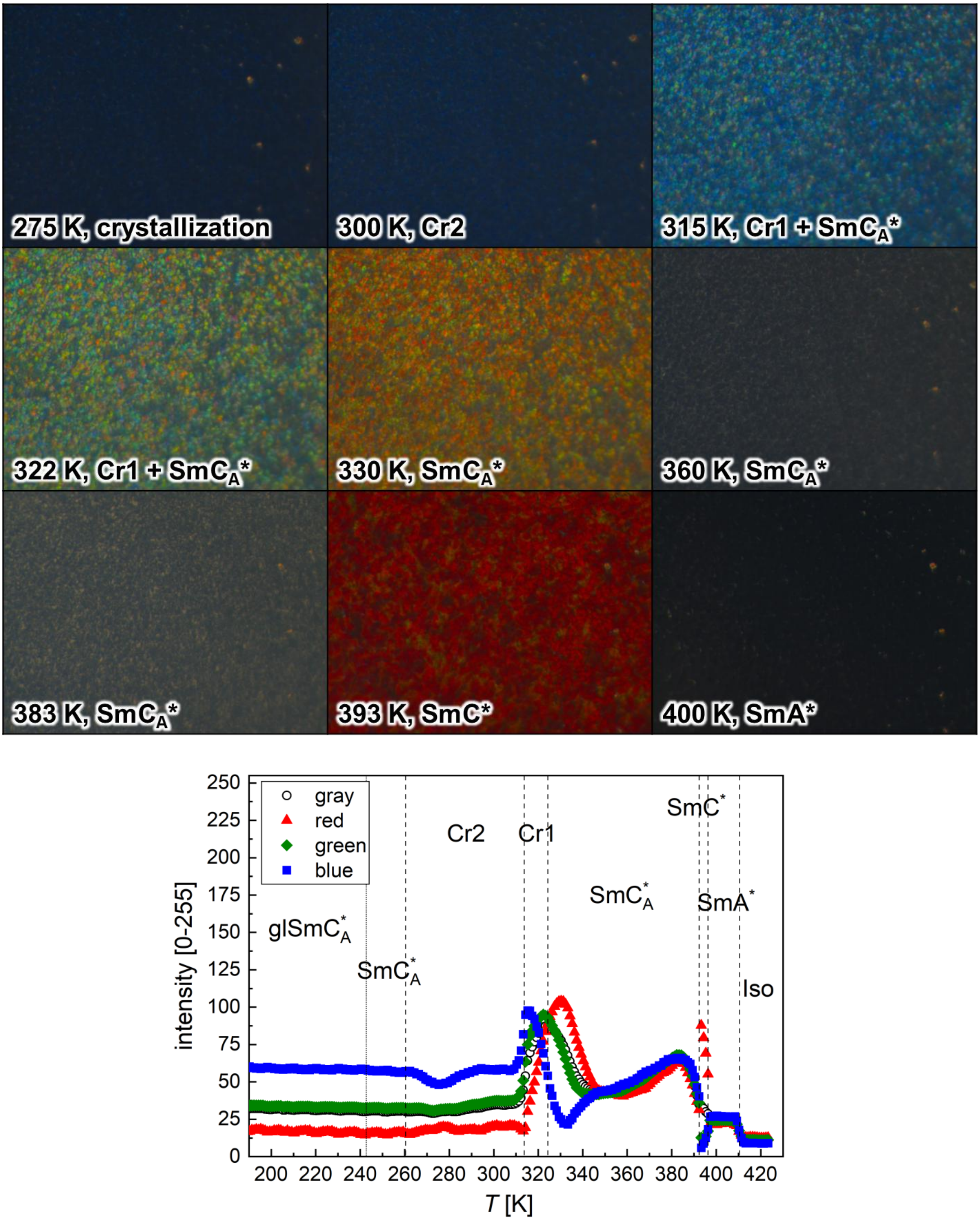

Figure S7. Representative POM textures (622 × 466 μm^2) of MIX5HFHH6 collected at the 10 K/min heating rate in the reflection mode as well as the red, green, blue components and weighted total intensity of each texture. The glass transition temperature is based on DSC results.

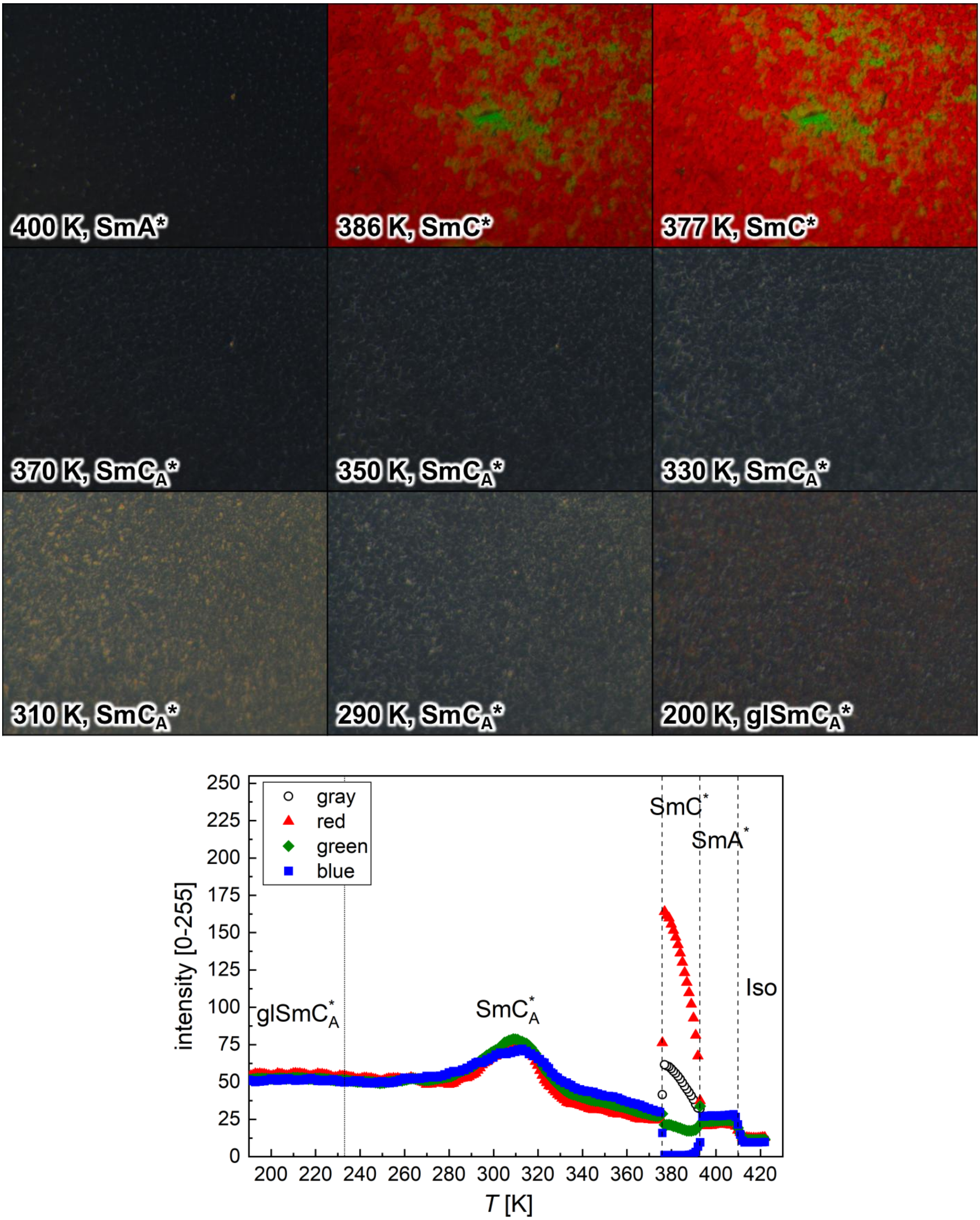

Figure S8. Representative POM textures (622 × 466 μm^2) of MIX6HFHH6 collected at the 10 K/min cooling rate in the reflection mode as well as the red, green, blue components and weighted total intensity of each texture. The glass transition temperature is based on DSC results.

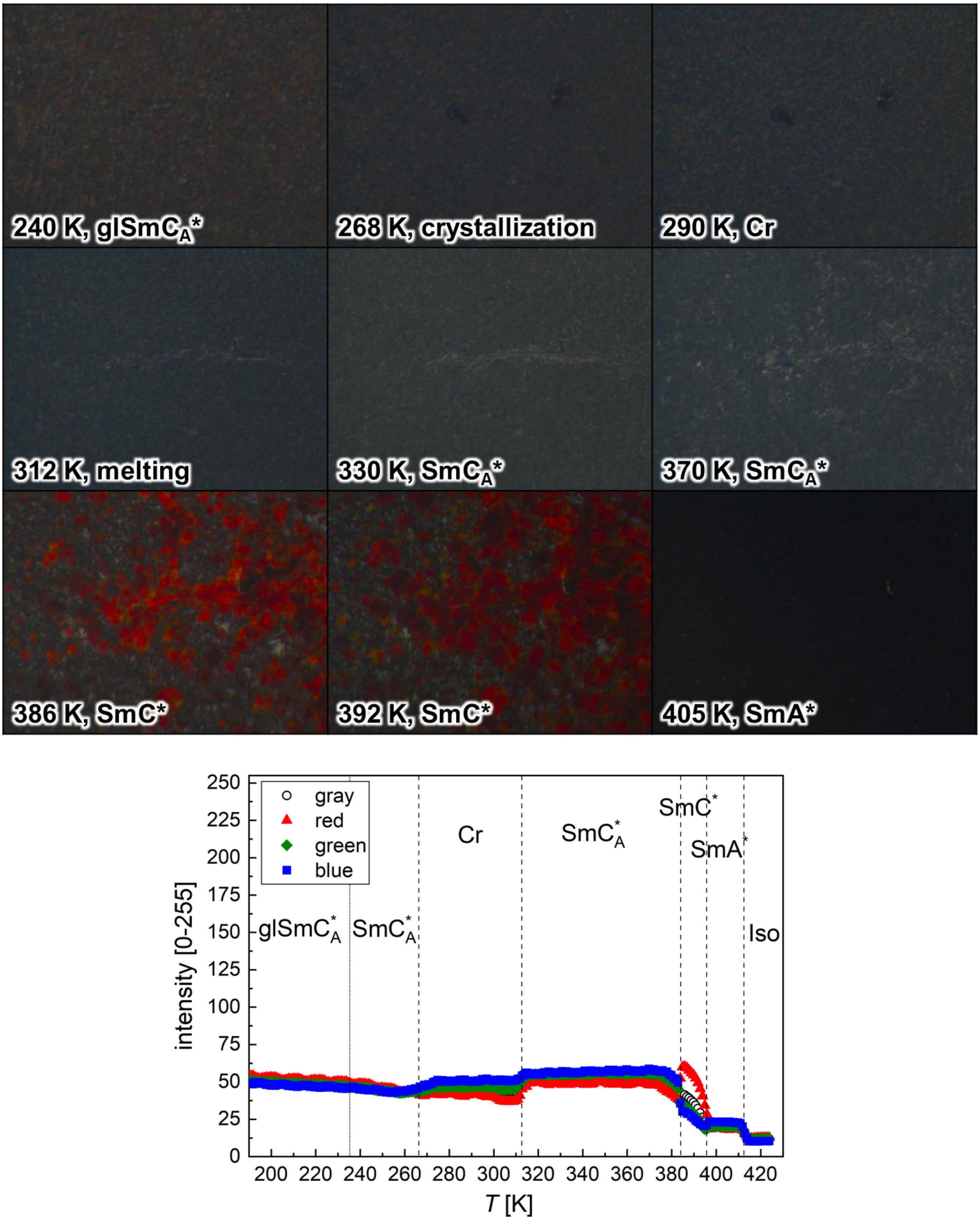

Figure S9. Representative POM textures (622 × 466 μm^2) of MIX6HFHH6 collected at the 10 K/min heating rate in the reflection mode as well as the red, green, blue components and weighted total intensity of each texture. The glass transition temperature is based on DSC results.

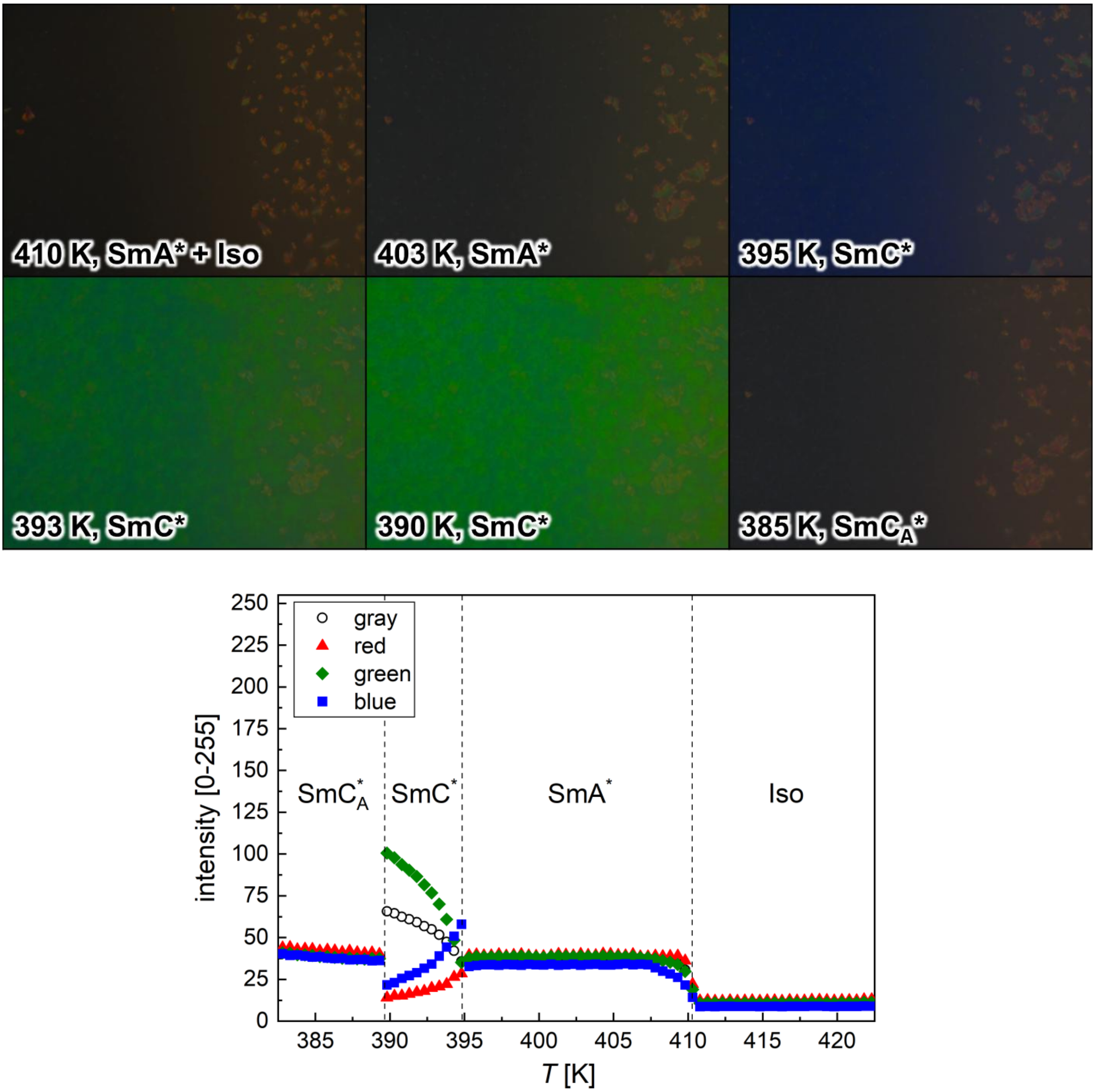

Figure S10. Representative POM textures (622 × 466 μm^2) of MIX5HFHH6 collected at the 2 K/min cooling rate in the reflection mode as well as the red, green, blue components and weighted total intensity of each texture.

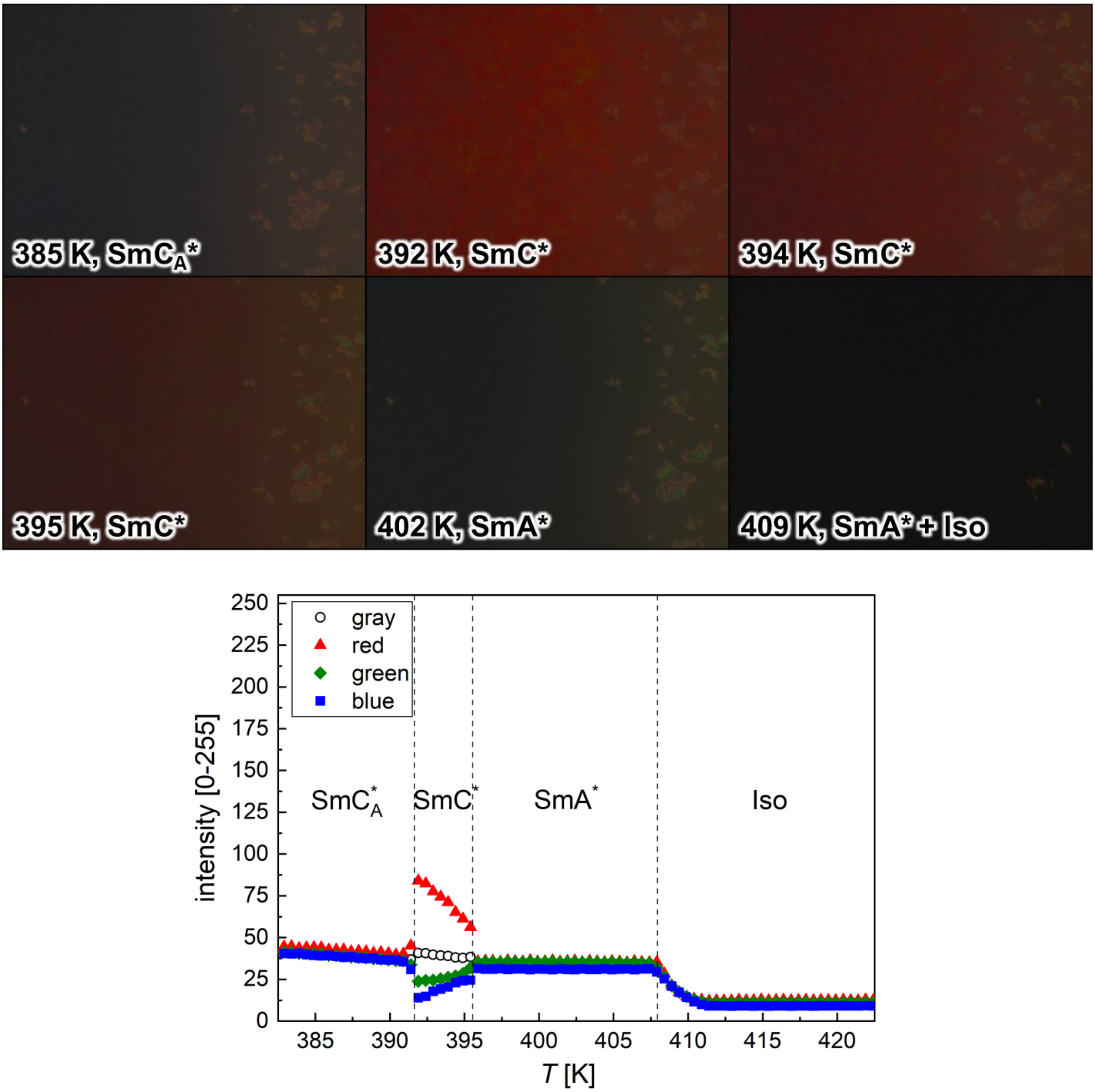

Figure S11. Representative POM textures (622 × 466 μm^2) of MIX5HFHH6 collected at the 2 K/min heating rate in the reflection mode as well as the red, green, blue components and weighted total intensity of each texture.

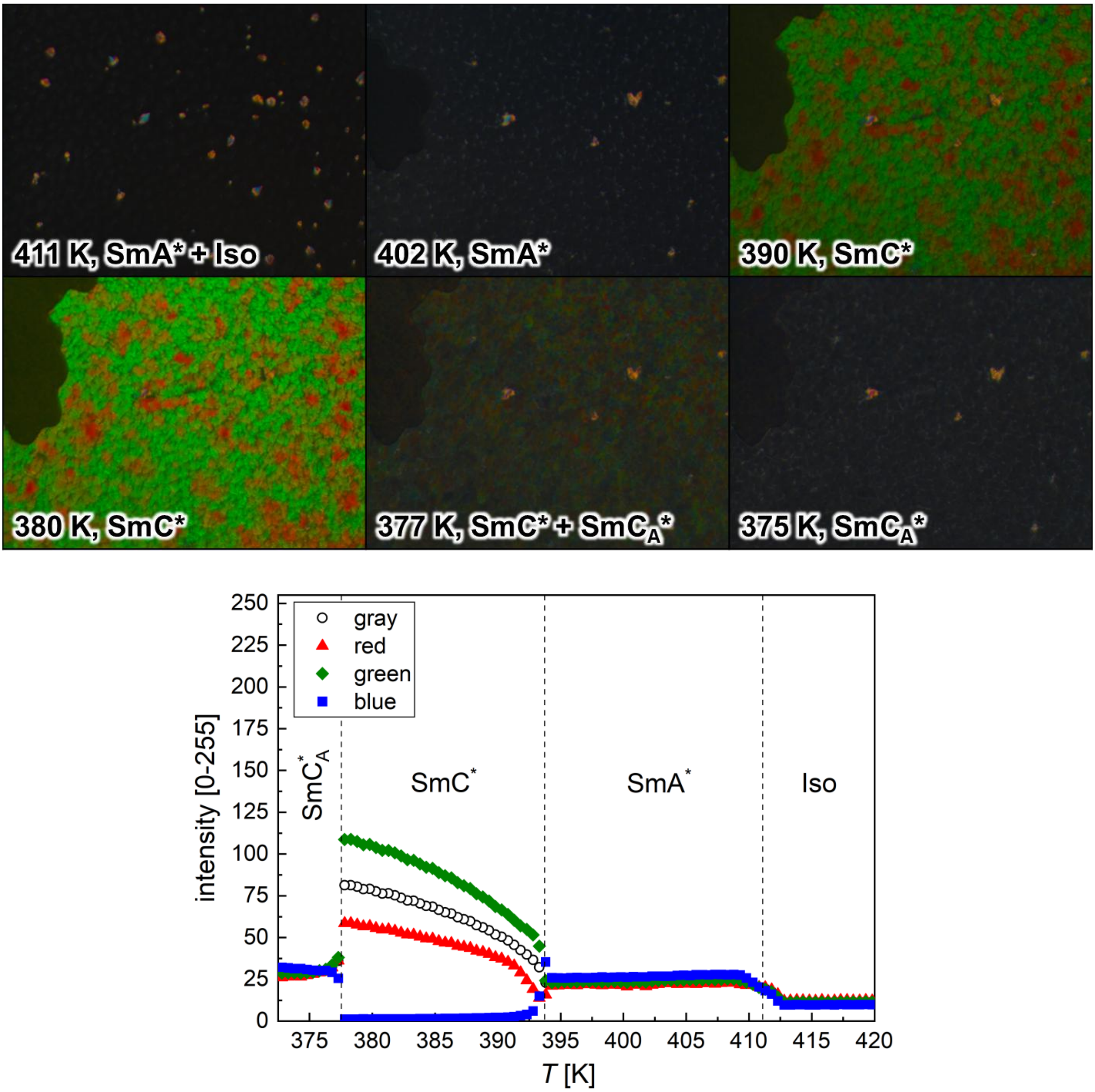

Figure S12. Representative POM textures (622 × 466 μm^2) of MIX6HFHH6 collected at the 2 K/min cooling rate in the reflection mode as well as the red, green, blue components and weighted total intensity of each texture.

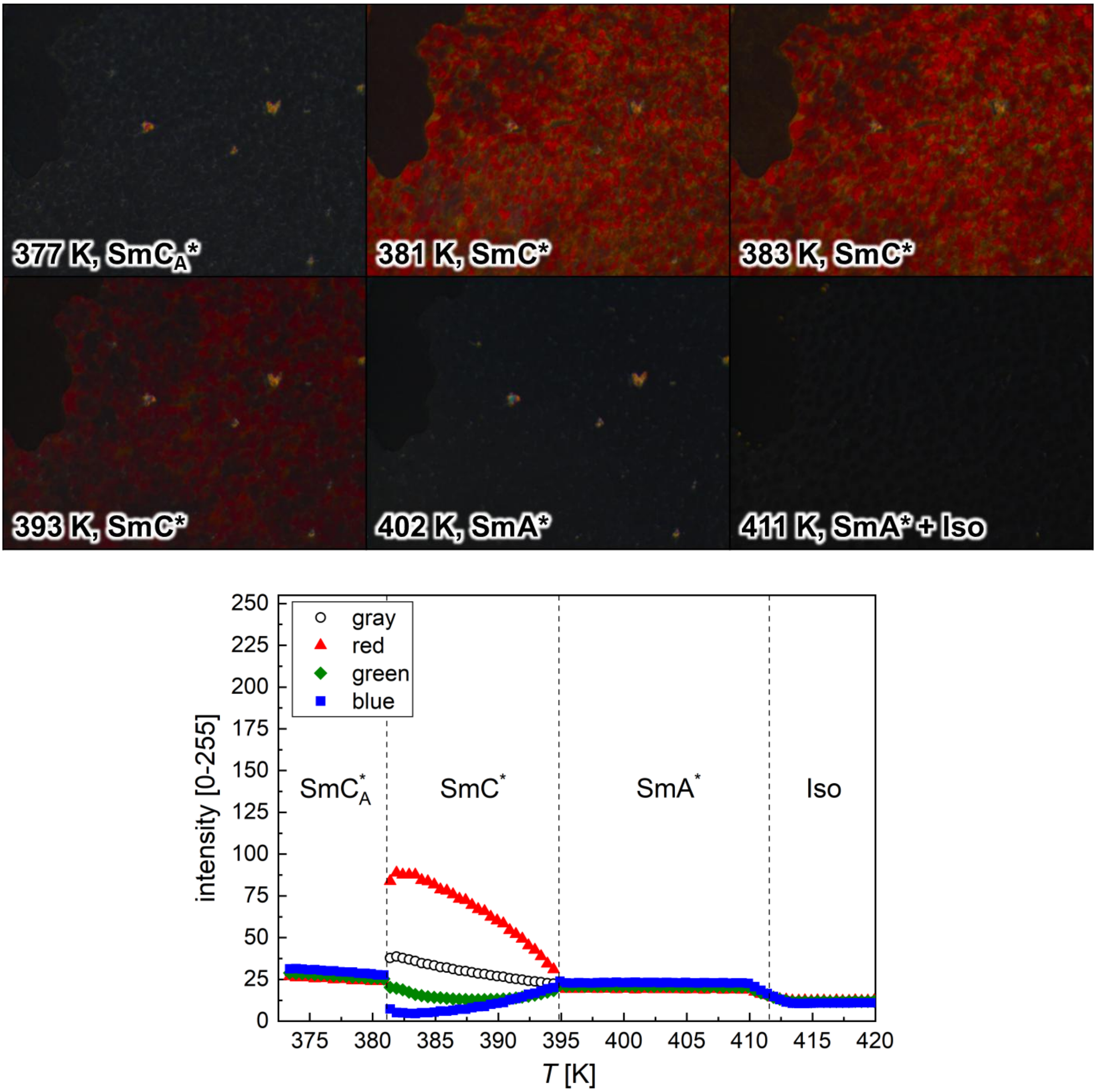

Figure S13. Representative POM textures (622 × 466 μm^2) of MIX6HFHH6 collected at the 2 K/min heating rate in the reflection mode as well as the red, green, blue components and weighted total intensity of each texture.

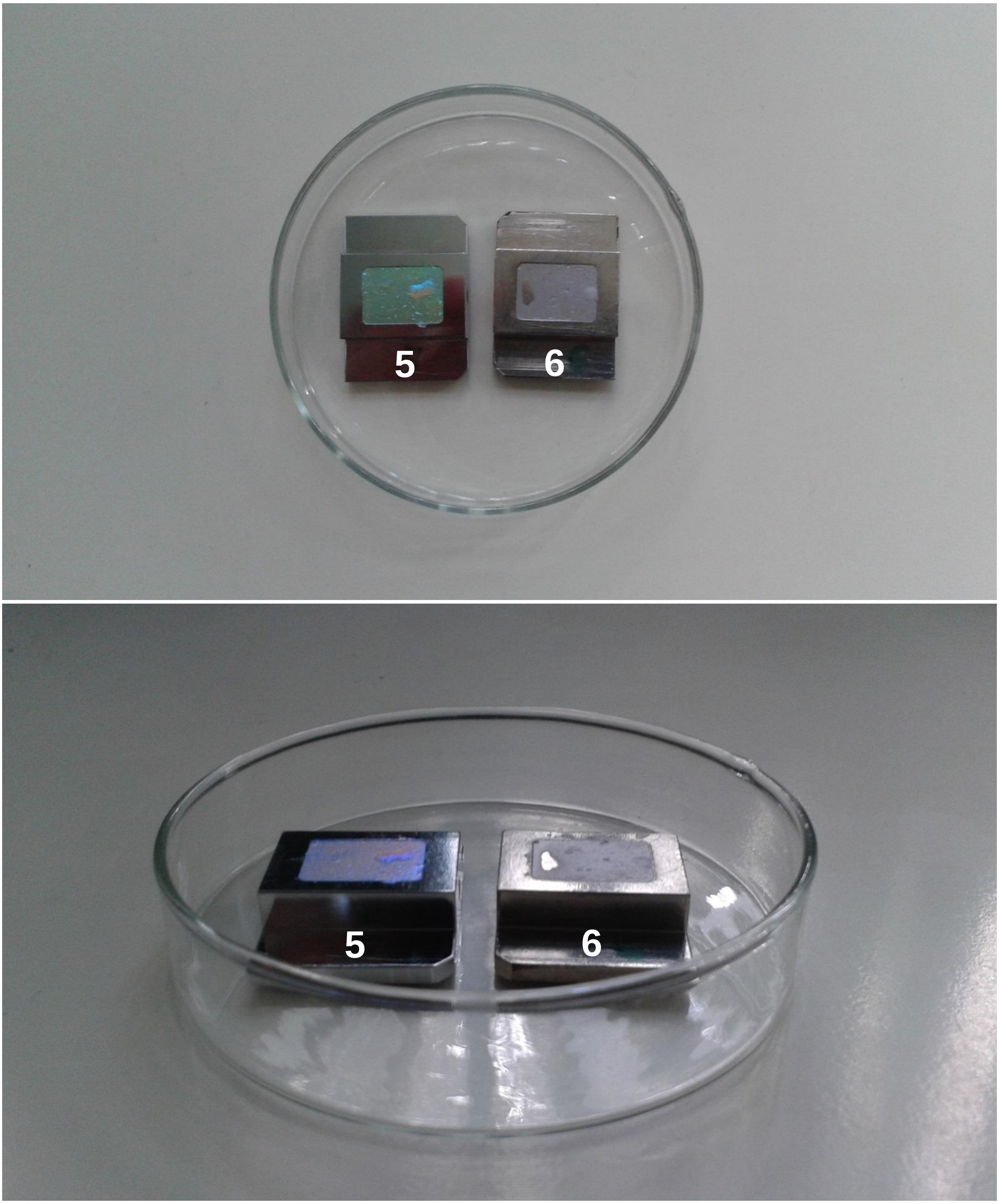

Figure S14. Homeotropically-aligned samples of MIXmHFHH6 (m = 5 on the left, m = 6 on the right) viewed from the top and from the side.

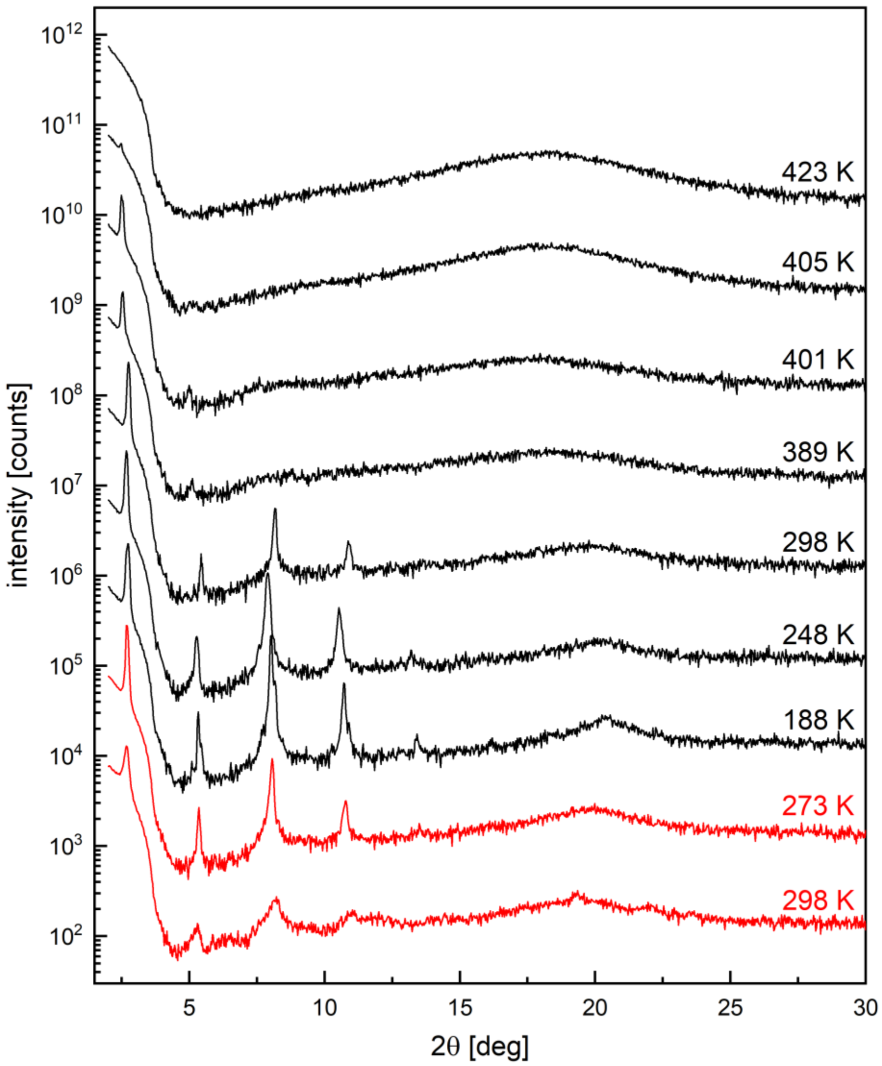

Figure S15. Selected XRD patterns of MIX5HFHH6 collected on cooling from 423 K to 188 K and on heating to 273 K and 298 K.

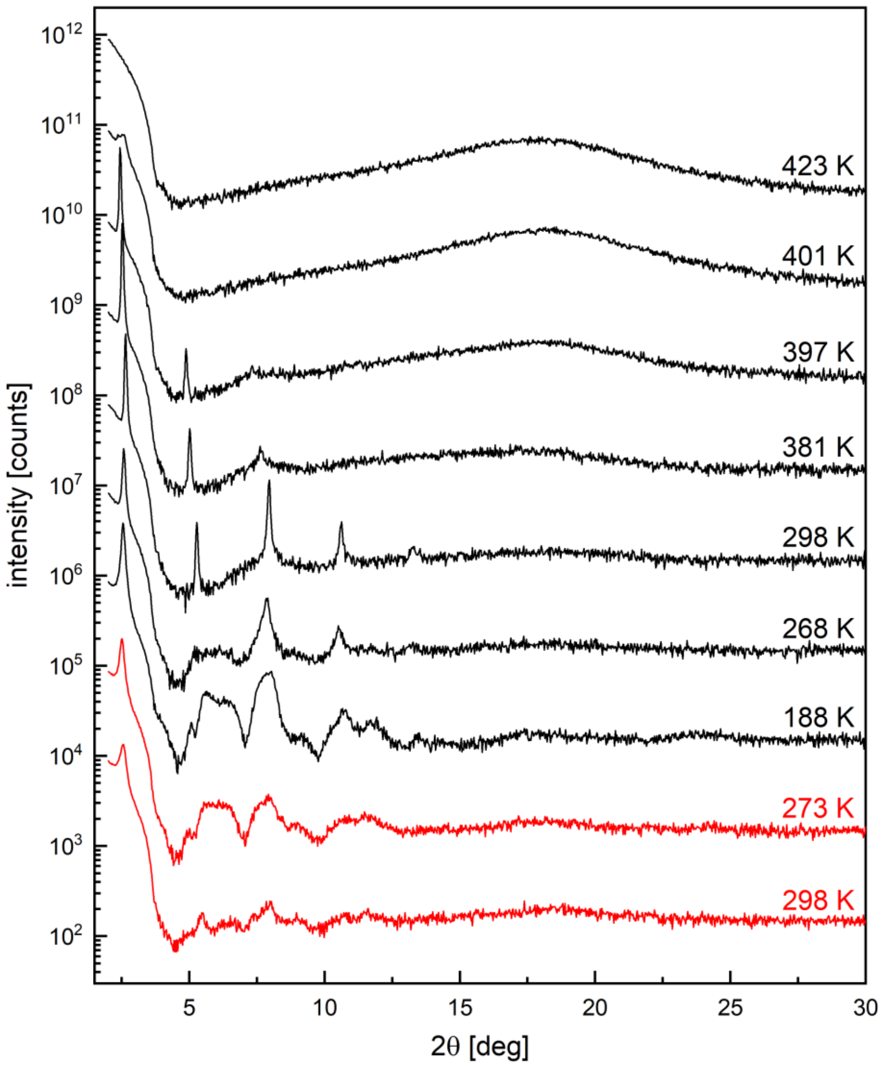

Figure S16. Selected XRD patterns of MIX6HFHH6 collected on cooling from 423 K to 188 K and on heating to 273 K and 298 K.
